# Channel Quality-Based Optimal Status Update for Information Freshness in Internet of Things

**DOI:** 10.3390/e23070912

**Published:** 2021-07-18

**Authors:** Fuzhou Peng, Xiang Chen, Xijun Wang

**Affiliations:** School of Electronics and Information Technology, Sun Yat-sen University, Guangzhou 510006, China; pengfzh@mail2.sysu.edu.cn (F.P.); wangxijun@mail.sysu.edu.cn (X.W.)

**Keywords:** age of information, status update, channel quality

## Abstract

This paper investigates the status updating policy for information freshness in Internet of things (IoT) systems, where the channel quality is fed back to the sensor at the beginning of each time slot. Based on the channel quality, we aim to strike a balance between the information freshness and the update cost by minimizing the weighted sum of the age of information (AoI) and the energy consumption. The optimal status updating problem is formulated as a Markov decision process (MDP), and the structure of the optimal updating policy is investigated. We prove that, given the channel quality, the optimal policy is of a threshold type with respect to the AoI. In particular, the sensor remains idle when the AoI is smaller than the threshold, while the sensor transmits the update packet when the AoI is greater than the threshold. Moreover, the threshold is proven to be a non-increasing function of channel state. A numerical-based algorithm for efficiently computing the optimal thresholds is proposed for a special case where the channel is quantized into two states. Simulation results show that our proposed policy performs better than two baseline policies.

## 1. Introduction

Recently, the Internet of things (IoT) has been widely used in the field of industrial manufacturing, environment monitoring, and home automation. In these applications, the sensors generate and transmit new status updates to the destination, where the freshness of the status updates is crucial for the destination to track the state of the environment and to make decisions. Thus, a new information freshness metric, namely age of information (AoI), was proposed in [1] to measure the freshness of updates from the receiver’s perspective. There are two widely used metrics, i.e., the average peak AoI [2] and the average AoI [3]. In general, the smaller the AoI is, the fresher the received updates are.

AoI was originally investigated in [1] for updating the status in vehicular networks. Considering the impact of the queueing system, the authors in [4] investigated the system performance under the M/M/1 and M/M/1/2 queueing systems with a first-come-first-served (FCFS) policy. Furthermore, the work of [5] studied how to keep the updates fresh by analyzing some general update policies, such as the zero-wait policy. The authors of [6] considered the optimal schedule problem for a more general cost that is the weighted sum of the transmission cost and the tracking inaccuracy for the information source. However, these works assumed that the communication channel is not error-prone. In practice, status updates are delivered through an erroneous wireless channel, which suffers from fading, interference, and noises. Therefore, the received updates may not be decoded correctly, which induces information aging and energy consumption.

There are several works that considered the erroneous channel [7,8]. The authors in [9] considered multiple communication channels and investigated the optimal coding and decoding schemes. The channel with an independent and identical packet error rate over time was considered in [10,11]. The work of [12] considered the impact of fading channels in packet transmission. A Markov channel was investigated in [13], where threshold policy was proven to be optimal, and a simulation-based approach was proposed to compute the corresponding threshold. However, how the information of channel quality should be exploited to improve system performance in information freshness remains to be investigated.

Channel quality indicator (CQI) feedback is commonly used in wireless communication systems [14]. In block fading channels, the channel quality, generally reported by the terminal, is highly relevant to the packet error rate (PER) [15] or, namely, the block error rate (BLER). It is probable that a received packet fails to be decoded when the channel suffers from a poor condition. However, a transmitter with the channel quality information is able to keep idle when there is deep fading, thereby saving energy. The channel quantization was also considered in [12,13], where the channel was quantized into multiple states. However, the decision making was not dependent on the channel state in [12], while [13] did not consider the freshness of information. These motivate us to introduce the information of channel quality into the design of the updating policy.

In this paper, a status update system with channel quality feedback is considered. In particular, the channel condition is quantized into multiple states, and the destination feeds the channel quality back to the sensor before the sensor updates the status. Our problem is to investigate the channel quality-based optimal status update policy, which minimizes the weighted sum of the AoI and the energy consumption. Our key contributions are summarized as follows:

- An average cost Markov decision process (MDP) is formulated to model this problem. Due to the infinite countable states and unbounded cost of the MDP, which makes analysis difficult, the discounted version of the original problem is first investigated, and the existence of the stationary and deterministic policy to the original problem is then proven. Furthermore, it is proven that the optimal policy is a threshold structure policy with respect to the AoI for each channel state by showing the monotonic property of the value function. We also prove that the threshold is a non-increasing function of channel state.
- By utilizing the threshold structure, a structure-aware policy iteration algorithm is proposed to efficiently obtain the optimal updating policy. Nevertheless, a numerical-based algorithm which directly computes the thresholds by non-linear fractional programming is also derived. Simulation results reveal the effects of system parameters and show that our proposed policy performs better than the zero-wait policy and periodic policy.

The rest of this paper is organized as follows. In Section 2, the system model is presented and the optimal updating problem is formulated. In Section 3, the optimal updating policy is proven to be of a threshold structure, and a threshold-based policy iteration algorithm is proposed to find the optimal policy. Section 4 presents the simulation results. Finally, we summarize our conclusions in Section 5.

## 2. System Model and Problem Formulation

### 2.1. System Description

**Figure 1:**
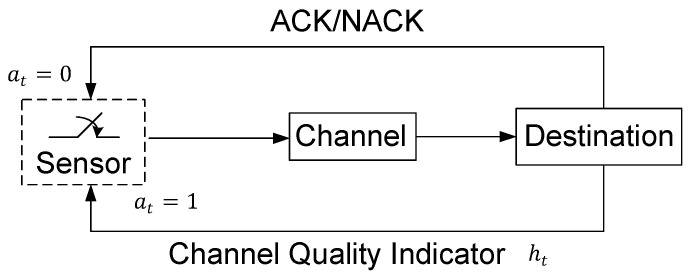
System model.

In this paper, we consider a status update system that consists of a sensor and a destination, as shown in Figure 1. Time is divided into slots. Without loss of generality, we assume that each time slot has an equal length, which is normalized to unity. At the beginning of each slot, the destination feeds the CQI back to the sensor. It is worth noting that the PER is different for different CQIs. Based on the CQI, the sensor decides in each time slot whether it should generate and transmit a new update to the destination via a wireless channel or keep idle for saving energy. These updates are crucial for the destination to estimate the states of the surrounding environment of the sensor and to make in-time decisions. Let $a_{t}$, which takes value from the action set A={0,1}, denote the action that the sensor performs in slot *t*, where $a_{t}=1$ means that the sensor generates and transmits a new update to the destination, and $a_{t}=0$ represents that the sensor is idle. If the sensor transmits an update packet in slot *t*, an acknowledgment will be fed back at the end of this time slot. In particular, an ACK is fed back when the destination successfully receives the update packet, and a NACK otherwise.

### 2.2. Channel Model

Suppose that the wireless channel is a block fading channel where the channel gain remains constant in each slot and varies independently over different slots. Let $z_{t}$ denote the channel gain in slot *t* which takes value from [0,+∞). We quantize the channel gain into N+1 levels which are denoted as $\left(z_{0},z_{1},\ldots ,z_{i},\ldots ,z_{N}\right)$. The quantization levels are arranged in an increasing order where $z_{0}=0$ and $z_{N}=\infty$. Hence, the channel is said to be in state *i* if the channel gain $z_{t}$ belongs to the interval $\left[z_{i},z_{i+1}\right)$. We denote by $h_{t}$ the state of the channel in slot *t*, where $h_{t}\in H≜\left\{0,1,2\ldots ,N-1\right\}$. With the aid of CQI fed back from the destination, the sensor has knowledge of the channel state at the beginning of each time slot.

Let $p_{z}\left(z\right)$ denote the distribution of the channel gain. Then, the probability of the channel being in state *i* is

(1) $$p_{i}=\int_{z_{i}}^{z_{i+1}}p_{z}\left(z\right)dz.$$

We assume that the signal-to-noise ratio (SNR) per information bit during the transmission remains constant. Then, the PER depends only on the channel gain. In particular, the PER for channel state *i* is given by

(2) $$g_{i}=\int_{z_{i}}^{z_{i+1}}P_{\mathrm{PER}}\left(z\right)p_{z}\left(z|i\right)dz,$$

where $P_{\mathrm{PER}}\left(z\right)$ is the PER of a packet with respect to the channel gain. The success probability $q_{i}$ of a packet transmitted over channel state *i* is $q_{i}=1-g_{i}$. According to [15], the success probability is a non-decreasing function of the channel state.

### 2.3. Age of Information

This paper uses the AoI as the freshness metric, which is defined as the time elapsed since the generation time of the latest update packet that is successfully received by the destination [1]. Let $G_{i}$ be the generation time of the *i*th successfully received update packet. Then, the AoI in time slot *t*, $\Delta_{t}$, is defined as

(3) $$\Delta_{t}=t-\max\left\{G_{i}:G_{i}\leq t\right\}.$$

In particular, if an update packet is successfully received, the AoI decreases to one. Otherwise, the AoI increases by one. Altogether, the evolution of the AoI is expressed by

(4) $$\Delta_{t+1}=\left\{\begin{array}{cc} 1, & \;\mathrm{if}\;\mathrm{the}\;\mathrm{transmission}\;\mathrm{is}\;\mathrm{successful},\\ \Delta_{t}+1, & \;\mathrm{otherwise}. \end{array}\right.$$

**Figure 2:**
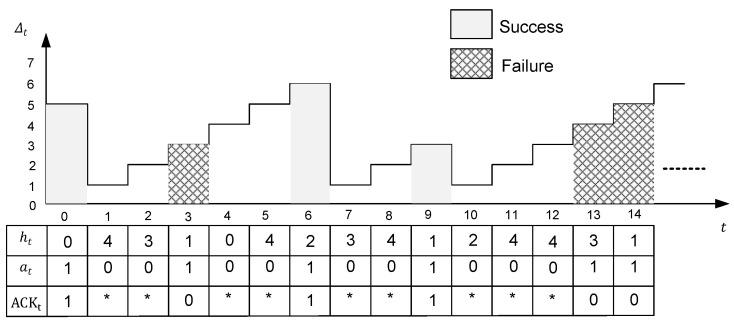
An example of the AoI evolution with the channel state $h_{t}$, the action $a_{t}$, and the acknowledgment ${\mathrm{ACK}}_{t}$. The asterisk stands for no acknowledgment from destination when the sensor keeps idle.

An example of the AoI evolution is shown in Figure 2, where the gray rectangle represents a successful reception of an update packet, and the mesh rectangle represents a transmission failure.

### 2.4. Problem Formulation

The objective of this paper is to find an optimal updating policy that minimizes the long-term average of the weighted sum of AoI and energy consumption. A policy π can be represented by the sequence of actions, i.e., $\pi =(a_{0},a_{1},\ldots ,a_{t},\ldots )$. Let Π be a set of stationary and deterministic policies. Then, the optimal updating problem is given by

(5) $$\min_{\pi \in \Pi }\underset{T\to \infty }{lim sup}\frac{1}{T}\sum_{t=0}^{T}E\left[\Delta_{t}+\omega a_{t}C_{e}\right],$$

where $C_{e}$ is the energy consumption, and ω is the weighting factor.

## 3. Optimal Updating Policy

This section aims to investigate the optimal updating policy for the problem formulated in above section. In this section, our investigating problem is first formulated into an infinite horizon average cost MDP, and the existence of a stationary and deterministic policy that minimizes the average cost is proven. Then, the non-decreasing property of the value function is derived. Based on this property, we prove that the optimal update policy is of a threshold structure with respect to AoI, and the optimal threshold is a non-increasing function of the channel state. Aiming to reduce the computational complexity, a structure-aware policy iteration algorithm is proposed to find the optimal policy. Moreover, non-linear fractional programming is employed to directly compute the optimal thresholds in a special case where the channel is quantized into two states.

### 3.1. MDP Formulation

The Markov decision process (MDP) is typically applied to address the optimal decision problem when the investigation problem can be characterized by the evolution of the system state and the cost is per-stage. The optimization problem in (5) can be formulated as an infinite horizon average cost MDP, which is elaborated in the following.

- States: The state of the MDP in slot *t* is defined as $x_{t}=\left(\Delta_{t},h_{t}\right)$, which takes values in $Z^{+}\times H$. Hence, the state space S is countable and infinite.
- Actions: The set of actions $a_{t}$ chosen in slot *t* is A={0,1}.
- Transition Probability: Let $\mathrm{Pr}(x_{t+1}|x_{t},a_{t})$ be the transition probability that the state $x_{t}$ in slot *t* transits to $x_{t+1}$ in slot t+1 after taking action $a_{t}$. According to the evolution of AoI in (4), the transition probability is given by
  (6) $$\left\{\begin{array}{c} \mathrm{Pr}\left(x_{t+1}=\left(\Delta +1,j\right)|x_{t}=\left(\Delta ,i\right),a_{t}=0\right)=p_{j},\\ \mathrm{Pr}\left(x_{t+1}=\left(1,j\right)|x_{t}=\left(\Delta ,i\right),a_{t}=1\right)=q_{i}p_{j},\\ \mathrm{Pr}\left(x_{t+1}=\left(\Delta +1,j\right)|x_{t}=\left(\Delta ,i\right),a_{t}=1\right)=g_{i}p_{j}. \end{array}\right.$$
- Cost: The instantaneous cost $C(x_{t},a_{t})$ at state $x_{t}$ given action $a_{t}$ in slot *t* is
  (7) $$C\left(x_{t},a_{t}\right)=\Delta_{t}+\omega a_{t}C_{e}.$$

For an MDP with infinite states and unbounded cost, it is not guaranteed to have a stationary and deterministic policy that attains the minimum average cost in general. Fortunately, we can prove the existence of stationary and deterministic policy in next sub-section.

### 3.2. The Existence of Stationary and Deterministic Policy

For rigorous mathematical analysis, this section is purposed to prove the existence of a stationary and deterministic optimal policy. According to [16], we first analyze the associated discounted cost problem of the original MDP. The expectation of discount cost with respect to discounted factor γ and initial state $\hat{x}$ under a policy π is given by

(8) $$V_{\pi ,\gamma }\left(\hat{x}\right)=E_{\pi }\left[\sum_{t=0}^{\infty }\gamma^{t}C\left(x_{t},a_{t}\right)|x_{0}=\hat{x}\right],$$

where $a_{t}$ is the decision made in state $\hat{x}$ under policy π, and γ∈(0,1) is the discounted factor. We first verify that $V_{\pi ,\gamma }\left(\hat{x}\right)$ is finite for any policy and all $\hat{x}\in S$.

**Lemma** **1.** *Given γ∈(0,1), for any policy π and all $\hat{x}=\left(\hat{x},\hat{h}\right)\in S$, we have*

(9) $$V_{\pi ,\gamma }\left(\hat{x}\right)=E_{\pi }\left[\sum_{t=0}^{\infty }\gamma^{t}C\left(x_{t},a_{t}\right)|x_{0}=\hat{x}\right]<\infty .$$

**Proof.**  By definition, the instantaneous cost in state $x_{t}=\left(\Delta_{t},h_{t}\right)$ given action $a_{t}$ is

(10) $$C\left(x_{t},a_{t}\right)=\left\{\begin{array}{cc} \Delta_{t}, & \mathrm{if}\;a_{t}=0,\\ \Delta_{t}+\omega C_{e}, & \mathrm{if}\;a_{t}=1. \end{array}\right.$$

Therefore, $C\left(x_{t},a_{t}\right)\leq \Delta_{t}+\omega C_{e}$ holds. Combined with the fact that the AoI increases, at most, linearly at each slot for any policy, we have

(11) $$\begin{array}{cc} \; & \sum_{t=0}^{\infty }\gamma^{t}C\left(x_{t},a_{t}|x_{0}=\left(\hat{\Delta },\hat{h}\right)\right)\\ \leq & \sum_{t=0}^{\infty }\gamma^{t}\left(\hat{\Delta }+t+\omega C_{e}\right)\\ = & \frac{1}{1-\gamma }\left(\hat{\Delta }+\frac{\gamma }{1-\gamma }+\omega \right)<\infty , \end{array}$$

which completes the proof.    □

Let $V_{\gamma }\left(\hat{x}\right)=\min_{\pi }V_{\pi ,\gamma }\left(\hat{x}\right)$ denote the minimum expected discounted cost. By Lemma 1, $V_{\gamma }\left(\hat{x}\right)=\min_{\pi }V_{\pi ,\gamma }\left(\hat{x}\right)<\infty$ holds for every $\hat{x}$ and γ∈(0,1).

According to [16] (Proposition 1), we have

(12) $$V_{\gamma }\left(\hat{x}\right)=\min_{a\in A}\left\{C\left(\hat{x},a\right)+\gamma \sum_{x^{\prime }\in S}\mathrm{Pr}\left(x^{\prime }|\hat{x},a\right)V_{\gamma }\left(x^{\prime }\right)\right\},$$

which implies that $V_{\gamma }\left(\hat{x}\right)$ satisfies the Bellman equation. $V_{\gamma }\left(\hat{x}\right)$ can be solved via a value iteration algorithm. In particular, we define $V_{\gamma ,0}\left(\hat{x}\right)=0$, and for all n≥1, we have

(13) $$V_{\gamma ,n}\left(\hat{x}\right)=\min_{a\in A}Q_{\gamma ,n}\left(\hat{x},a\right),$$

where

(14) $$Q_{\gamma ,n}\left(\hat{x},a\right)=\min_{a\in A}\left\{C\left(\hat{x},a\right)+\gamma \sum_{x^{\prime }\in S}\mathrm{Pr}\left(x^{\prime }|\hat{x},a\right)V_{\gamma ,n-1}\left(x^{\prime }\right)\right\}$$

is related to the right-hand-side (RHS) of the discounted cost optimality equation. Then, $\lim_{n\to \infty }V_{\gamma ,n}\left(\hat{x}\right)=V_{\gamma }\left(\hat{x}\right)$ for every $\hat{x}$ and γ.

Now, we can use the value iteration algorithm to establish the monotonic properties of $V_{\gamma }\left(\hat{x}\right)$

**Lemma** **2.** *For all *Δ* and i, we have*

(15) $$V_{\gamma }\left(\Delta ,N-1\right)\leq V_{\gamma }\left(\Delta ,i\right),$$

*and for all $\Delta_{1}\leq \Delta_{2}$ and i, we have*

(16) $$V_{\gamma }\left(\Delta_{1},i\right)\leq V_{\gamma }\left(\Delta_{2},i\right).$$

**Proof.**  See Appendix A.    □

Based on Lemmas 1 and 2, we are ready to show that the MDP has a stationary and deterministic optimal policy in the following theorem.

**Theorem** **1.** For the MDP in (5), there exists a stationary and deterministic optimal policy $\pi^{*}$ that minimizes the long-term average cost. Moreover, there exists a finite constant $\lambda =\lim_{\gamma \to 1}\left(1-\gamma \right)V_{\gamma }\left(x\right)$ for all states x, where λ is independent of the initial state, and a value function V(x), such that

(17) $$\lambda +V\left(x\right)=\min_{a\in A}\left\{C\left(x,a\right)+\sum_{x^{\prime }\in S}\mathrm{Pr}\left(x^{\prime }|x,a\right)V\left(x^{\prime }\right)\right\}$$

*holds for all x.*

**Proof.**  See Appendix B.    □

### 3.3. Structural Analysis

According to Theorem 1, the optimal policy for the average cost problem satisfies the following equation

(18) $$\pi^{*}\left(x\right)=\arg\min_{a\in A}Q\left(x,a\right),$$

where

(19) $$Q\left(x,a\right)=C\left(x,a\right)+\sum_{x^{\prime }\in S}\mathrm{Pr}\left(x^{\prime }|x,a\right)V\left(x^{\prime }\right).$$

Similar to Lemma 2, the monotonic property of the value function V(x) is given in the following lemma.

**Lemma** **3.** *Given the channel state i, for any $\Delta_{2}\geq \Delta_{1}$, we have*

(20) $$V\left(\Delta_{2},i\right)\geq V\left(\Delta_{1},i\right).$$

**Proof.**  This proof follows the same procedure of Lemma 2, with one exception being that the value iteration algorithm is based on Equation (17).    □

Moreover, based on Lemma 3, the property of the increment of the value function is established in following lemma.

**Lemma** **4.** *Given the channel state i, for any $\Delta_{2}\geq \Delta_{1}$, we have*

(21) $$V\left(\Delta_{2},i\right)-V\left(\Delta_{1},i\right)\geq \Delta_{2}-\Delta_{1}.$$

**Proof.**  We first examine the relation between the state-action value functions, i.e., $Q(\Delta_{2},i,a)$ and $Q(\Delta_{1},i,a)$. Specifically, based on Lemma 3, we have

(22) $$\begin{array}{ll} & Q\left(\Delta_{2},i,0\right)-\left(\Delta_{2}-\Delta_{1}\right)=\Delta_{1}+\sum_{j=0}^{N-1}p_{j}V\left(\Delta_{2}+1,j\right)\\ \geq & \Delta_{1}+\sum_{j=0}^{N-1}p_{j}V\left(\Delta_{1}+1,j\right)=Q\left(\Delta_{1},i,0\right), \end{array}$$

and

(23) $$\begin{array}{cc} \; & Q\left(\Delta_{2},i,1\right)-\left(\Delta_{2}-\Delta_{1}\right)\\ = & \Delta_{1}+\omega C_{e}+q_{i}\sum_{j=0}^{N-1}p_{j}V\left(1,j\right)+g_{i}\sum_{j=0}^{N-1}p_{j}V\left(\Delta_{2}+1,j\right)\\ \geq & \Delta_{1}+\omega C_{e}+q_{i}\sum_{j=0}^{N-1}p_{j}V\left(1,j\right)+g_{i}\sum_{j=0}^{N-1}p_{j}V\left(\Delta_{1}+1,j\right)\\ = & Q(\Delta_{1},i,1). \end{array}$$

Since $V\left(x\right)=\min_{a\in A}Q\left(x,a\right)$, we complete the proof.    □

Our main result is presented in the following theorem.

**Theorem** **2.** For any given channel state i, there exists a threshold $\beta_{i}$, such that when $\Delta \geq \beta_{i}$, the optimal action is to generate and transmit a new update, i.e., $\pi^{*}\left(\Delta ,i\right)=1$, and when $\Delta <\beta_{i}$, the optimal action is to remain idle, i.e., $\pi^{*}\left(\Delta ,i\right)=0$. Moreover, the optimal threshold $\beta_{i}$ is a non-increasing function of channel state i, i.e., $\beta_{i}\geq \beta_{j}$ holds for all i,j∈H and i≤j.

**Proof.**  See Appendix C.    □

According to Theorem 2, the sensor will not update the status until the AoI exceeds the threshold. Moreover, if the channel condition is not good, i.e., channel state *i* is small, the sensor will wait for a longer time before it samples and transmits the status update packet so as to reduce the energy consumption because of a higher probability of transmission failure.

Based on the threshold structure, we can reduce the computational complexity of the policy iteration algorithm to find the optimal policy. The details of the algorithm are presented in Algorithm 1.
**Algorithm 1** Policy iteration algorithm (PIA) based on the threshold structure.1:Initialization: Set k=0, iteration threshold ϵ, and initialize the value function $V_{0}\left(x\right)=0$ and policy π(x)=0 for all state x∈S2:**repeat**3: k←k+1.4:  Based on last iterative value function $V_{k-1}\left(x\right)$, compute the current value function $V_{k}\left(x\right)$ by calculating the following equations.5:$\;V_{k}\left(x\right)=\min_{a\in A}\left\{C\left(x,a\right)+\sum_{x^{\prime }\in S}\mathrm{Pr}\left(x^{\prime }|x,a\right)V_{k-1}\left(x^{\prime }\right)\right\}$6:**until**$|V_{k}\left(x\right)-V_{k-1}\left(x\right)|\leq \epsilon$ for all x∈S7:**for**x=(Δ,i)∈S**do**8:  **if** $x^{\prime }=\left(\Delta -1,i\right)\in S$ and $\pi (x^{\prime })=1$ **then**9:  π(x)←1.10: **else**11:$\;\pi \left(x\right)\leftarrow \arg\min_{a\in A}\left\{C\left(x,a\right)+\underset{x^{\prime }\in S}{\mathrm{Pr}}\left(x^{\prime }|x,a\right)V\left(x^{\prime }\right)\right\}$12: **end if**13:**end for**14:$\pi^{*}\leftarrow \pi$15:**return** the optimal policy $\pi^{*}$


### 3.4. Computing the Thresholds for a Special Case

**Figure 3:**
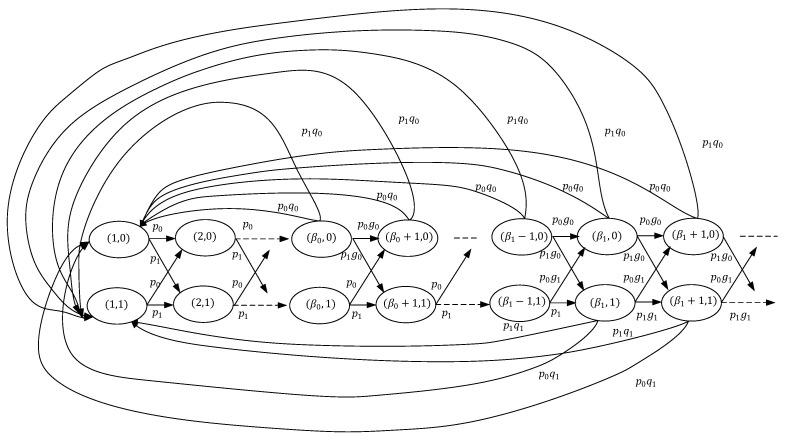
An illustration of established Markov chain with two channel states.

In the above section, we have proven that the optimal policy has a threshold structure. Given the thresholds $\left(\beta_{0},\beta_{1},\ldots ,\beta_{N-1}\right)$, a Markov chain can be induced by the threshold policy. A special Markov chain is depicted in Figure 3, where the channel has two states. By leveraging the Markov chain, we first derive the average cost of the special case, which is summarized in the following theorem.

**Theorem** **3.** *Let φ(x) be the steady state probability of state x of the corresponding Markov chain with two states and $\beta_{0},\beta_{1}$ be the threshold with respect to the channel state, respectively. The steady state probability is given by*

(24) $$\varphi \left(i,j\right)=\left\{\begin{array}{cc} p_{j}\varphi_{1}, & \mathrm{if}\;1\leq i\leq \beta_{1},\\ p_{j}s_{0}^{i-\beta_{1}}\varphi_{1}, & \mathrm{if}\;\beta_{1}<i\leq \beta_{0},\\ p_{j}s_{0}^{\beta_{0}-\beta_{1}}s_{1}^{i-\beta_{1}}\varphi_{1}, & \mathrm{if}\;i>\beta_{0}, \end{array}\right.$$

*where $\varphi_{1}=\varphi \left(1,0\right)+\varphi \left(1,1\right)$, $s_{0}=1-p_{1}q_{1}$, $s_{1}=1-p_{0}q_{0}-p_{1}q_{1}$, and $\varphi_{1}$ satisfies following equation:*

(25) $$\varphi_{1}=\frac{1}{\beta_{1}+\frac{s_{0}-s_{0}^{\beta_{0}-\beta_{1}}}{1-s_{0}}+s_{0}^{\beta_{0}-\beta_{1}}\frac{s_{1}}{1-s_{1}}}.$$

*The average cost then is given by*

(26) $$\begin{array}{cc} \; & C_{mc}\left(\beta_{0},\ldots ,\beta_{N-1}\right)\\ = & \varphi_{1}\left(\frac{\beta_{1}\left(\beta_{1}+1\right)}{2}+A+B+\omega C_{e}E\right), \end{array}$$

*where*

(27) $$A=\frac{s_{0}\left(\left(\beta_{1}+1\right)-\beta_{0}s_{0}^{\beta_{0}-\beta_{1}}\right)}{1-s_{0}}+\frac{s_{0}^{3}-s_{0}^{\beta_{0}-\beta_{1}+1}}{{\left(1-s_{0}\right)}^{2}},$$

(28) $$B=\frac{\left(\beta_{0}+1\right)s_{1}}{1-s_{1}}+\frac{s_{1}^{3}}{1-s_{1}},$$

*and*

(29) $$\begin{array}{c} E=\left(s_{0}^{\beta_{0}-\beta_{1}}\frac{1}{1-s_{1}}+p_{1}\frac{1-s_{0}^{\beta_{0}-\beta_{1}}}{1-s_{0}}\right). \end{array}$$

**Proof.**  See Appendix D.    □

Therefore, the closed form of the average cost is a function of thresholds. By linear search or gradient descent algorithm, the numerical solution of optimal thresholds can be obtained. However, computing its gradient directly requires a large amount of computation till convergence. Here, a nonlinear fractional programming (NLP) [17] based algorithm which can efficiently obtain the numerical solution is proposed.

Let $x=(\beta_{0},\beta_{1})$. We can rewrite the cost function as a fractional form, where the numerator is denoted as $N\left(x\right)=-C_{mc}\left(x\right)/\varphi_{1}$, and the denominator term is $N\left(x\right)=1/\varphi_{1}$. The solution to an NLP problem with the form in the following

(30) $$\max\left\{\frac{N(x)}{D(x)}|x\in A\right\}$$

is related to the optimization problem (31)

(31) $$\max\left\{N(x)-qD(x)|(x\in A\right\},\;\mathrm{for}\;q\in R,$$

where the following assumption should also be satisfied:

(32) D(x)>0,forallx∈A.

Define the function F(q) with variable *q* as

(33) $$F\left(q\right)=\max\left\{N(x)-qD(x)|x\in A\right\},\;\mathrm{for}\;q\in R.$$

According to [17], F(q) is a strictly monotonic decreasing function and is convex over R. Furthermore, we have $q_{0}=N\left(x_{0}\right)/D\left(x_{0}\right)=\max\left\{N\left(x\right)-qD\left(x\right)|x\in A\right\}$ if, and only if,

(34) $$F\left(q_{0}\right)=\max\left\{N\left(x\right)-q_{0}D\left(x\right)|x\in A\right\}=0.$$

Then, the algorithm can be described by two steps. The first step is to solve a convex optimization problem with a one dimensional parameter by a bisection method. The second step is to solve a high dimensional optimization problem by a gradient descent method.

According to [17], a bisection method can be used to solve the optimal $q_{0}$, under the assumption that the value of function F(q) can be obtained exactly for given *q*. We will actually use the gradient descent algorithm to obtain the numerical solution of F(q) since the global search method may not perform in polynomial time. As a trick, we alternate the optimization variables of thresholds $\left(\beta_{0},\beta_{1}\right)$ by the variables of the decrement of thresholds, i.e., $x=(\beta_{0}-\beta_{1},\beta_{1})$. To summarize, the numerical-based method for computing the optimal thresholds is given by Algorithm 2.
**Algorithm 2** Numerical computation of the optimal thresholds.**Input:**Iterationtimek,errorthresholdδ**Output:**$\mathrm{Numerical}\;\mathrm{result}\;x^{*}$1:Let $N\left(x\right)=-C_{mc}\left(x\right)/\varphi_{1}$, and $D\left(x\right)=1/\varphi_{1}$. Define F(q)=max{N(x)−qD(x)|x≥0}2:Let the iteration starts with i=1, search range [a,b] of *q*.3:**while**i≤k**do**4:$\;m=\frac{a+b}{2}$;5:**  if**F(m)∗F(a)<0**then**6:  b=m;7:**  else**8:  a=m;9:**  end if**10:** if**$\frac{b-a}{2}<\delta$**then**11:$\;x^{*}=\arg\min_{x}F\left(m\right)$12:   break;13:** end if**14:   i=i+1;15:**end while**


## 4. Simulation Results and Discussions

In this section, the simulation results are presented to investigate the impacts of the system parameters. We also compare the optimal policy with the zero-wait policy and periodic policy, where the zero-wait policy immediately generates an update at each time slot and the periodic policy keeps a constant interval between two updates.

**Figure 4:**
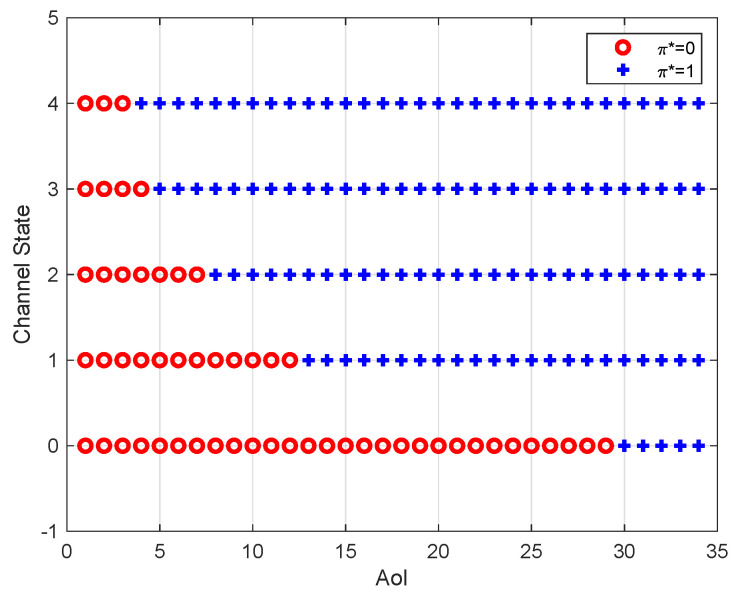
Optimal policy for different AoI and channel states ($q_{0}=0.1,\;q_{1}=0.2,\;q_{2}=0.3,\;q_{3}=0.4,\;q_{4}=0.5,\;p_{0}=0.1,\;p_{1}=0.1,\;p_{2}=0.3,\;p_{3}=0.3,\;p_{4}=0.2,\;\omega =10,\;C_{e}=1$).

Figure 4 depicts the optimal policy for different AoI and channel states, where the number of channel states is 5. It can be seen that, for each channel state, the optimal policy has a threshold structure with respect to the AoI. In particular, when the AoI is small, it is not beneficial for the sensor to generate and transmit a new update because the energy consumption dominates the total cost. We can also see that the threshold is non-increasing with the channel state. In other words, if the channel condition is better, the threshold is smaller. This is because the success probability of packet transmission increases with the channel state.

**Figure 5:**
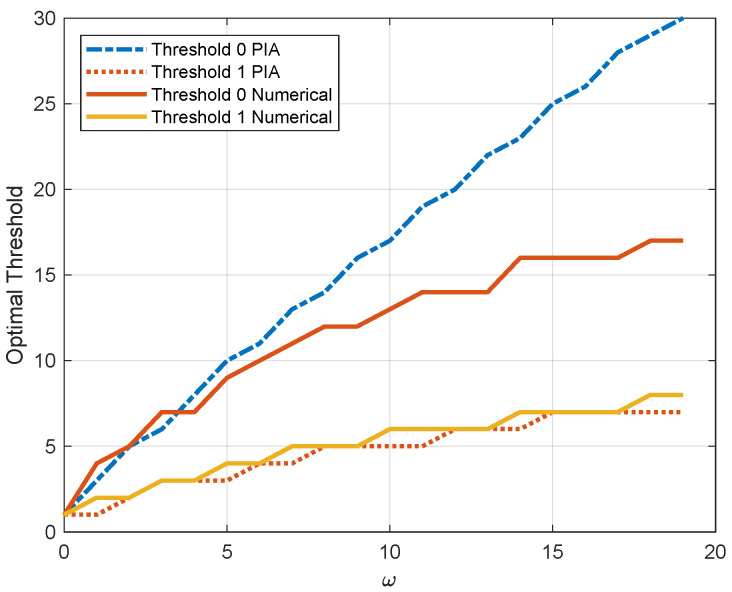
Optimal thresholds for two different channel states versus ω ($p_{0}=0.2,\;p_{1}=0.8,\;q_{0}=0.2,\;q_{1}=0.5,\;C_{e}=1$).

Figure 5 illustrates the thresholds for the MDP with two channel states with respect to the weighting factor ω, in which the two dashed lines are obtained by PIA and the other two solid lines are obtained by the proposed numerical algorithm. Both of the thresholds grow with the increasing of ω. Since the energy consumption has more weight, it is not efficient to update when the AoI is small. On the contrary, when ω decreases, the AoI dominates and the thresholds decline. In particular, both of the thresholds equal 1 when ω=0. In this case, the optimal policy reduces to the zero-wait policy. We can also see that the value of the threshold for channel state 1 of the numerical algorithm is close to the optimal solution. In contrast, the value of the threshold for channel state 0 gradually deviates from the optimal value.

**Figure 6:**
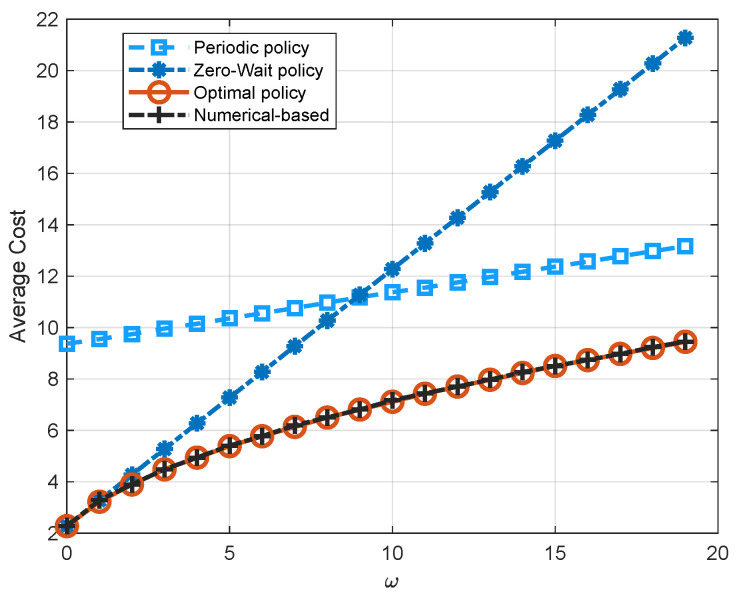
Comparison of the zero-wait policy, the periodic policy with period being 5, the numerical-based policy, and the optimal policy with respect to the weighting factor ω ($p_{0}=0.2,\;p_{1}=0.8,\;q_{0}=0.2,\;q_{1}=0.5,\;C_{e}=1$).

Figure 6 illustrates the performance comparison of four policies, i.e., the zero-wait policy, the periodic policy, the numerical-based policy, and the optimal policy, with respect to the weighting factor ω. It is easy to see that the optimal policy has the lowest average cost. As we see in Figure 6, the zero-wait policy has the same performance with the optimal policy when ω=0. As ω increases, the average cost of all three policies increases. However, the increment of the zero-wait policy is larger than the periodic policy and the optimal policy due to the frequent transmission in the zero-wait policy. Although the thresholds obtained by the PIA and the numerical algorithm are not exactly the same as shown in Figure 5, the performance of the numerical-based algorithm also coincides with the optimal policy. This is because the threshold for channel state 1 exists in the quadratic term of the cost function, while the threshold for channel state 0 exists in the negative exponential term of the cost function. As a result, the threshold for channel state 1 has a much more significant effect on the system performance.

**Figure 7:**
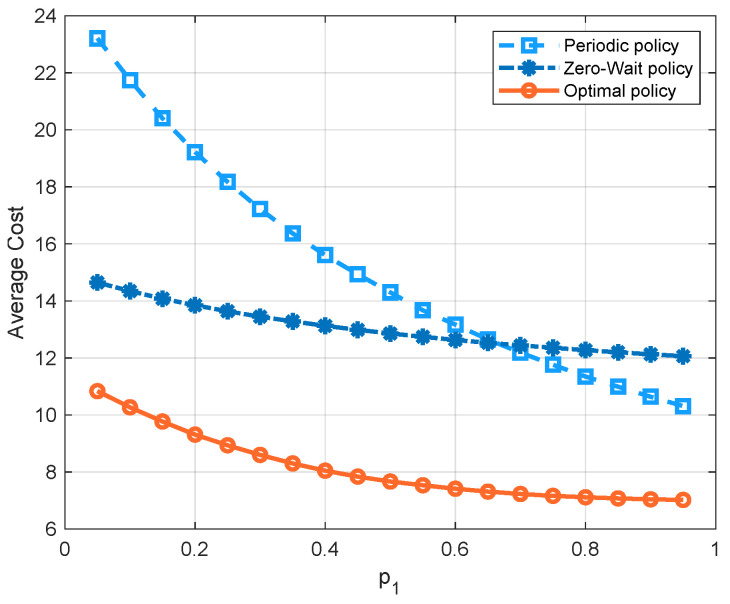
Comparison of the zero-wait policy, the periodic policy with period being 5, and the optimal policy with respect to $p_{1}$ ($q_{0}=0.2,\;q_{1}=0.5,\;\omega =10,\;C_{e}=1$).

**Figure 8:**
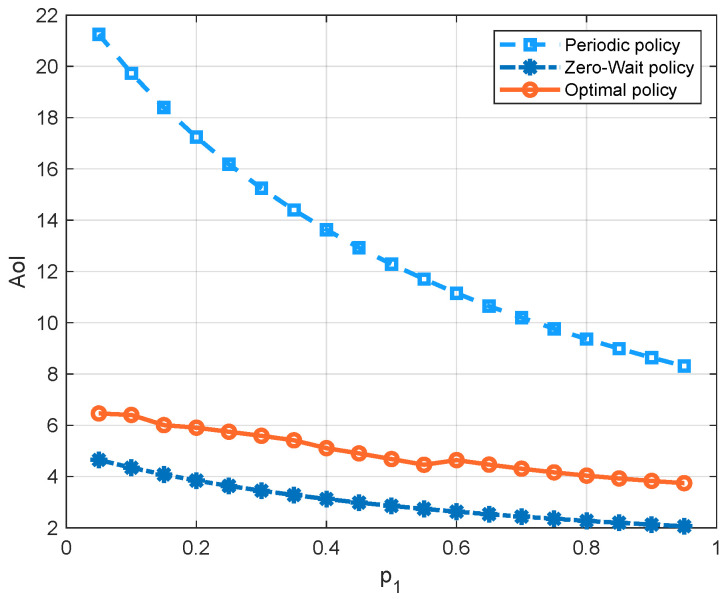
AoI comparison of the zero-wait policy, the periodic policy with period being 5, and the optimal policy with respect to $p_{1}$ ($q_{0}=0.2,\;q_{1}=0.5,\;\omega =10,\;C_{e}=1$).

**Figure 9:**
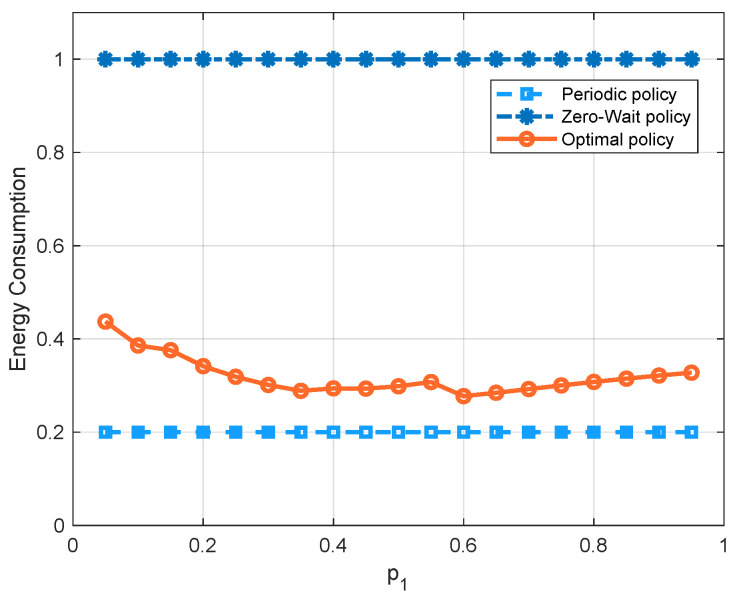
Energy consumption comparison of the zero-wait policy, the periodic policy with period being 5, and the optimal policy with respect to $p_{1}$ ($q_{0}=0.2,\;q_{1}=0.5,\;\omega =10,\;C_{e}=1$).

Figure 7 compares the three policies with respect to the probability $p_{1}$ of the channel being in state 1. Since there is a higher probability that the channel has a good quality as $p_{1}$ increases, the average cost of all three policies decreases. We can see that, in the regime of $p_{1}$, the optimal policy has the lowest average cost, because it can achieve a good balance between the AoI and the energy consumption. We can also see that the cost of the periodic policy is greater than the zero-wait policy first, and smaller later. To further demonstrate these curves, we separate the energy consumption term and AoI term into different figures, i.e., Figure 8 and Figure 9. We see that the update cost of the zero-wait policy is smaller than that of the periodic policy, but the AoI of the zero-wait policy has a smaller decrease with respect to $p_{1}$ than the periodic policy.

## 5. Conclusions

In this paper, we have studied the optimal updating policy in an IoT system, where the channel gain is quantized into multiple states and the channel state is fed back to the sensor before the decision making. The status update problem has been formulated as an MDP to minimize the long-term average of the weighted sum of the AoI and the energy consumption. By investigating the properties of the value function, it is proven that the optimal policy has a threshold structure with respect to AoI for any given channel state. We have also proven that the threshold is a non-increasing function of the channel state. Simulation results show the impacts of system parameters on the optimal thresholds and the average cost. Through comparisons, we have also shown that our proposed policy outperforms the zero-wait policy and the periodic policy. In our future research, the time-varying channel model will be further involved for guiding the future design of realistic IoT systems.

## Data Availability

Not applicable.

## Appendix A. Proof of Lemma 2

Based on the value iteration algorithm, the induction method can be employed in following proof. Firstly, we initial that $V_{\gamma ,0}\left(x\right)=0$, where both Equations (15) and (16) hold for all x∈S.

### Appendix A.1. Proof of Equation (16)

When n=1,

(A1) $$\begin{array}{cc} \; & Q_{\gamma ,1}\left(\Delta_{1},i,0\right)-Q_{\gamma ,1}\left(\Delta_{2},i,0\right)=\Delta_{1}-\Delta_{2}\leq 0, \end{array}$$

and

(A2) $$\begin{array}{cc} \; & Q_{\gamma ,1}\left(\Delta_{1},i,1\right)-Q_{\gamma ,1}\left(\Delta_{2},i,1\right)=\Delta_{1}+\omega C_{e}-\left(\Delta_{2}+\omega C_{e}\right)\leq 0, \end{array}$$

hold due to $\Delta_{1}\leq \Delta_{2}$, and we have $V_{\gamma ,1}\left(\Delta_{1},i\right)\leq V_{\gamma ,1}\left(\Delta_{2},i\right)$.

Suppose that $V_{\gamma ,K}\left(\Delta_{1},i\right)\leq V_{\gamma ,K}\left(\Delta_{2},i\right)$ holds for k≤K. Considering the case of k=K+1,

(A3) $$\begin{array}{cc} \; & Q_{\gamma ,K+1}\left(\Delta_{1},i,0\right)-Q_{\gamma ,K+1}\left(\Delta_{2},i,0\right)=\Delta_{1}-\Delta_{2}\leq 0, \end{array}$$

and

(A4) $$\begin{array}{cc} \; & Q_{\gamma ,K+1}\left(\Delta_{1},i,1\right)-Q_{\gamma ,K+1}\left(\Delta_{2},i,1\right)\\ = & \left(\Delta_{1}-\Delta_{2}\right)+\gamma \sum_{j\in H}p_{j}g_{i}\left(V_{\gamma ,K}\left(\Delta_{1}+1,j\right)-V_{\gamma ,K}\left(\Delta_{2}+1,j\right)\right)\\ \leq & 0, \end{array}$$

hold for all *i* according to $\Delta_{1}\leq \Delta_{2}$. Therefore, we have $V_{\gamma ,K+1}\left(\Delta_{1},i\right)\leq V_{\gamma ,K+1}\left(\Delta_{2},i\right)$. Since $\lim_{n\to \infty }V_{\gamma ,n}=V_{\gamma }$, we have $V_{\gamma }\left(\Delta_{1},i\right)\leq V_{\gamma }\left(\Delta_{2},i\right)$.

### Appendix A.2. Proof of Equation (15)

By the definition of function $Q_{\gamma }\left(x,a\right)$, we have

(A5) $$Q_{\gamma }\left(\Delta ,i,0\right)=\Delta +\gamma \sum_{j\in H}p_{j}V_{\gamma }\left(\Delta +1,j\right),$$

and

(A6) $$\begin{array}{cc} \; & Q_{\gamma }\left(\Delta ,i,1\right)=\Delta +\omega C_{e}+\gamma \left(\sum_{j\in H}p_{j}\left(g_{i}V_{\gamma }\left(\Delta +1,j\right)+q_{i}V_{\gamma }\left(1,j\right)\right)\right). \end{array}$$

Therefore,

(A7) $$\begin{array}{c} Q_{\gamma }\left(\Delta ,N-1,0\right)-Q_{\gamma }\left(\Delta ,i,0\right)\leq 0, \end{array}$$

and

(A8) $$\begin{array}{cc} \; & Q_{\gamma }\left(\Delta ,N-1,1\right)-Q_{\gamma }\left(\Delta ,i,1\right)\\ \overset{\left(a\right)}{=} & \gamma \sum_{j\in H}p_{j}\left(q_{N-1}-q_{i}\right)\left(V_{\gamma }\left(1,j\right)-V_{\gamma }\left(\Delta +1,j\right)\right)\leq 0, \end{array}$$

hold for all *i*, where step (a) is due to Equation (16). Hence, we have $V_{\gamma }\left(\Delta ,N-1\right)\leq V_{\gamma }\left(\Delta ,i\right)$. This completes the whole proof.

## Appendix B. Proof of Theorem 1

Theorem 1 can be proven by verifying the conditions given in [16]. The conditions are listed as follows:

- (1): For every state x and discount factor γ, the discount value function $V_{\gamma }\left(x\right)$ is finite.
- (2): There exists a non-negative value *L* such that $-L\leq h_{\gamma }\left(x\right)$ for all x and γ, where $h_{\gamma }\left(x\right)=V_{\gamma }\left(x\right)-V_{\gamma }\left(\hat{x}\right)$, and $\hat{x}$ is a reference state.
- (3): There exists a non-negative value $M_{x}$, such that $h_{\gamma }\left(x\right)\leq M_{x}$ for every x and γ. For every x, there exists an action $a_{x}$ such that $\sum_{x^{\prime }}\mathrm{Pr}\left(x^{\prime }|x,a_{x}\right)M_{x^{\prime }}<\infty$.
- (4): The inequality $\sum_{x^{\prime }}\mathrm{Pr}\left(x^{\prime }|x,a\right)M_{x^{\prime }}<\infty$ holds for all x and *a*.

By Lemma 1, $V_{\gamma }\left(\hat{x}\right)=\min_{\pi }V_{\pi ,\gamma }\left(\hat{x}\right)<\infty$ holds for every $\hat{x}$ and γ. Hence, condition (1) holds. According to Lemma 2, by letting $\hat{x}=\left(1,N-1\right)$ and L=0, we have $h_{\gamma }\left(x\right)\geq 0$, which verifies condition (2).

Before verifying condition (3), a lemma is given as follows:

**Lemma** **A1.**
*Let us denote $\hat{x}=\left(1,N-1\right)$ as the reference state and define the first time that an initial state x transits to $\hat{x}$ as $K=\min\left\{k:k\leq 1,x_{k}=\hat{x}\right\}$. Then, the expectation cost under the always-transmitting policy $\pi_{a}$, i.e., the sensor generates and transmits a new update in each slot, is*

(A9) $$\begin{array}{cc} C_{x,\hat{x}}\left(\pi_{a}\right)= & E_{\pi_{a}}\left[\sum_{t=0}^{K-1}\gamma^{t}C\left(x_{t},a_{t}\right)|x\right], \end{array}$$

*where $C_{x,\hat{x}}\left(\pi_{a}\right)<\infty$ holds for all x.*

**Proof.** Since $a_{t}=1$ for all *t*, the probability that the state returns to $\hat{x}$ from x after exactly *K* slot is given by

(A10) $$\begin{array}{cc} \; & \mathrm{Pr}\left(K=k|x=\left(\Delta ,j\right)\right)=\left\{\begin{array}{cc} p_{N-1}q_{j}, & \mathrm{if}\;k=1,\\ p_{N-1}g_{j}{\left(1-\sum_{i\in H}p_{i}q_{i}\right)}^{k-2}\left(\sum_{i\in H}p_{i}q_{i}\right), & \mathrm{otherwise}. \end{array}\right. \end{array}$$

Then, the expectation return cost from x to $\hat{x}$ is expressed as

(A11) $$\begin{array}{cc} \; & C_{x,\hat{x}}\left(\pi_{a}\right)\\ = & E\left[\sum_{t=0}^{K-1}\gamma^{t}C\left(x_{t},a_{t}\right)|x\right]\\ \overset{\left(a\right)}{\leq } & \sum_{k=1}^{\infty }\mathrm{Pr}\left(K=k|x=(\Delta ,j)\right)\left[\sum_{m=0}^{k-1}\left(\Delta +m+\omega C_{e}\right)\right]\\ < & \infty , \end{array}$$

where step (a) is due to the fact that $C\left(x_{t},a_{t}\right)\leq \Delta_{t}+\omega C_{e}$. □

Considering a mixture policy π, in which it performs the always-transmitting policy $\pi_{a}$ from initial state x until it enters the reference state $\hat{x}$, it later performs the optimal policy $\pi_{\gamma }$ that minimizes the discounted cost. Therefore, we have

(A12) $$\begin{array}{cc} \; & V_{\gamma }\left(x\right)\\ \leq & E_{\pi_{a}}\left[\sum_{t=0}^{K-1}\gamma^{t}C\left(x_{t},a_{t}\right)|x\right]+E_{\pi_{b}}\left[\sum_{t=K}^{\infty }\gamma^{t}C\left(x_{t},a_{t}\right)|\hat{x}\right]\\ \leq & C_{x,\hat{x}}\left(\pi_{a}\right)+E_{\pi }\left[\gamma^{K}V_{\gamma }\left(\hat{x}\right)\right]\\ \leq & C_{x,\hat{x}}\left(\pi_{a}\right)+V_{\gamma }\left(\hat{x}\right), \end{array}$$

which implies that $h_{\gamma }\left(x\right)\leq C_{x,\hat{x}}\left(\pi_{a}\right)$. Hence, let $\hat{x}=\left(1,N-1\right)$ and $M_{x}=C_{x,\hat{x}}\left(\pi_{a}\right)$; condition (3) is verified.

On the other hand, $M_{x}<\infty$ holds for all x. The states that transit from x are finite. Thus, the weighted sum of finite $M_{x}$ is also finite, i.e., $\sum_{x^{\prime }}P\left(x^{\prime }|x,a\right)M_{x^{\prime }}<\infty$ holds for all x and *a*, which verifies condition (4). This completes the whole verification.

## Appendix C. Proof of Theorem 2

Based on the definition of Q(Δ,i,a), we can obtain the difference between the state-action value function as follows:

(A13) $$\begin{array}{cc} \; & Q(\Delta ,i,0)-Q(\Delta ,i,1)\\ = & \sum_{j=0}^{N-1}p_{j}V\left(\Delta +1,j\right)-q_{i}\sum_{j=0}^{N-1}p_{j}V\left(1,j\right)-g_{i}\sum_{j=0}^{N-1}p_{j}V\left(\Delta +1,j\right)-\omega C_{e}\\ = & q_{i}\sum_{j=0}^{N-1}p_{j}\left(V(\Delta +1,j)-V(1,j)\right)-\omega C_{e}\\ \overset{\left(a\right)}{\geq } & q_{i}\Delta -\omega C_{e}. \end{array}$$

where (a) is due to the property of the value function given in Lemma 4. We then discuss the difference between the state-action value function in two cases.

Case 1: ω=0.

In this case, Q(Δ,i,0)−Q(Δ,i,1)≥0 holds for any Δ and *i*. Therefore, the optimal policy is to update at each slot in spite of the channel state. In other words, the optimal thresholds are all equal to 1.

Case 2: ω>0.

We note that, given *i*, $q_{i}\Delta -\omega C_{e}$ increases linearly with Δ. Hence, there exists a positive integer ${\hat{\beta }}_{i}$, such that ${\hat{\beta }}_{i}$ is the minimum value that satisfies $q_{i}{\hat{\beta }}_{i}-\omega C_{e}\geq 0$. Therefore, if $\Delta \geq {\hat{\beta }}_{i}$, $Q\left(\Delta ,i,0\right)-Q\left(\Delta ,i,1\right)\geq q_{i}{\hat{\beta }}_{i}-\omega C_{e}\geq 0$ holds. This implies that there must exist a threshold $\beta_{i}$ satisfying $1\leq \beta_{i}\leq {\hat{\beta }}_{i}$. If $\Delta \geq \beta_{i}$, we have Q(Δ,i,0)−Q(Δ,i,1)≥0.

Altogether, the optimal policy has a threshold structure for ω≥0. Then, we examine the non-increasing property of the thresholds. Firstly, we show that the difference between the state-action value function is monotonic with respect to the channel state by fixing the AoI. Assuming that i,j∈H and i≤j, it is easy to obtain that

(A14) $$\begin{array}{cc} \; & Q(\Delta ,j,0)-Q(\Delta ,j,1)-(Q(\Delta ,i,0)-Q(\Delta ,i,1))\\ = & \left(q_{j}-q_{i}\right)\sum_{l=0}^{N-1}p_{l}\left(V\left(\Delta +1,l\right)-V\left(1,l\right)\right)\geq 0. \end{array}$$

Since Q(Δ,i,0)−Q(Δ,i,1)≥0 when $\Delta \geq \beta_{i}$, we have Q(Δ,j,0)−Q(Δ,j,1)≥0 according to (A14). This implies that the optimal threshold $\beta_{j}$ corresponding to channel state *j* is no greater than $\beta_{i}$, i.e., $\beta_{j}\leq \beta_{i}$. This completes the whole proof.

## Appendix D. Proof of Theorem 3

Assume that φ(x) is the steady probability of state x in a Markov chain. The steady state probability φ(x) satisfies the following global balance equation [18], i.e.,

(A15) $$\varphi \left(x\right)=\sum_{x^{\prime }\in S}\varphi \left(x^{\prime }\right)\mathrm{Pr}\left(x|x^{\prime }\right).$$

Let $\varphi_{1}=\varphi \left(1,0\right)+\varphi \left(1,1\right)$. We prove Equation (A23) by discussing three cases via mathematical induction.

Case 1: $1<i\leq \beta_{1}$

Based on Equation (A15), we have

(A16) $$\begin{array}{cc} \varphi (2,j)= & \varphi \left(1,0\right)p_{j}+\varphi \left(1,1\right)p_{j}\\ = & p_{j}\varphi_{1}. \end{array}$$

Assuming that $\varphi \left(i,j\right)=p_{j}\varphi_{1}$ holds for all $i\leq k<\beta_{1}$, we examine φ(k+1,j). We have

(A17) $$\begin{array}{cc} \varphi (k+1,j)= & \varphi \left(k,0\right)p_{j}+\varphi \left(k,1\right)p_{j}\\ = & p_{j}p_{0}\varphi_{1}+p_{j}p_{1}\varphi_{1}\\ = & p_{j}\varphi_{1}, \end{array}$$

which completes this segment of the proof.

Case 2: $\beta_{1}<i\leq \beta_{0}$

Similarly, we have

(A18) $$\begin{array}{cc} \varphi (\beta_{1}+1,j)= & p_{j}\left(1-q_{1}\right)\varphi \left(\beta_{1},1\right)+p_{j}\varphi \left(\beta_{1},0\right)\\ = & p_{j}\varphi_{1}\left(\left(1-q_{1}\right)p_{1}+p_{0}\right)\\ = & p_{j}\varphi_{1}s_{0}, \end{array}$$

where $s_{0}=1-p_{1}q_{1}$. Assuming that $\varphi \left(i,j\right)=p_{j}\varphi_{1}s_{0}^{i-\beta_{1}}$ holds for all $\beta_{1}<i\leq k<\beta_{0}$, we examine φ(k+1,j). We have

(A19) $$\begin{array}{cc} \varphi (k+1,j)= & p_{j}\left(1-q_{1}\right)\varphi \left(k,1\right)+p_{j}\varphi \left(k,0\right)\\ = & p_{j}\varphi_{1}\left(\left(1-q_{1}\right)p_{1}+p_{0}\right)s_{0}^{k-\beta_{1}}\\ = & p_{j}\varphi_{1}s_{0}^{k+1-\beta_{1}}, \end{array}$$

which completes this segment of the proof.

Case 3: $i>\beta_{0}$

Follow above discussion, we have

(A20) $$\begin{array}{cc} \varphi (\beta_{0}+1,j)= & p_{j}\left(1-q_{1}\right)\varphi \left(\beta_{0},1\right)+p_{j}\left(1-q_{0}\right)\varphi \left(\beta_{0},0\right)\\ = & p_{j}\varphi_{1}s_{0}^{\beta_{0}-\beta_{1}}\left(\left(1-q_{1}\right)p_{1}+\left(1-q_{0}\right)p_{0}\right)\\ = & p_{j}\varphi_{1}s_{0}^{\beta_{0}-\beta_{1}}s_{1}, \end{array}$$

where $s_{1}=1-p_{0}q_{0}-p_{1}q_{1}$. Assuming that $\varphi \left(i,j\right)=p_{j}\varphi_{1}s_{0}^{\beta 0-\beta_{1}}s_{1}^{i-\beta_{0}}$ holds for all $\beta_{0}<i\leq k$, we examine φ(k+1,j). We have

(A21) $$\begin{array}{cc} \; & \varphi (k+1,j)\\ = & p_{j}\left(1-q_{1}\right)\varphi \left(k,1\right)+p_{j}\left(1-q_{0}\right)\varphi \left(k,0\right)\\ = & p_{j}\varphi_{1}s_{0}^{\beta_{0}-\beta_{1}}\left(\left(1-q_{1}\right)p_{1}+\left(1-q_{0}\right)p_{0}\right)s_{1}^{k-\beta_{0}}\\ = & p_{j}\varphi_{1}s_{0}^{\beta_{0}-\beta_{1}}s_{1}^{k+1-\beta_{0}}. \end{array}$$

Altogether, we obtain the steady state probability with respect to an unknown parameter $\varphi_{1}$. According to the fact that $\sum_{i=1}^{\infty }\sum_{j=0}^{N-1}\varphi \left(i,j\right)=1$, we formulate an equation:

(A22) $$\begin{array}{cc} \; & \sum_{i=1}^{\infty }\sum_{j=0}^{N-1}\varphi \left(i,j\right)\\ = & \varphi_{1}\left\{\beta_{1}+\sum_{i=\beta_{1}+1}^{\beta_{0}}s_{0}^{i-\beta_{1}}+\sum_{i=\beta_{0}+1}^{\infty }s_{0}^{\beta_{0}-\beta_{1}}s_{1}^{i-\beta_{0}}\right\}\\ = & \varphi_{1}\left\{\beta_{1}+\frac{s_{0}-s_{0}^{\beta_{1}-\beta_{0}}}{1-s_{0}}+s_{0}^{\beta_{1}-\beta_{0}}\frac{s_{1}}{1-s_{1}}\right\}\\ = & 1, \end{array}$$

where the expression of $\varphi_{1}$ is obtained.

The average cost of a Markov chain is given by

(A23) $$C_{mc}=\sum_{x\in S}\varphi \left(x\right)C\left(x,\pi^{*}\left(x\right)\right).$$

Substituting (24) into (A23), we have

$$\begin{array}{cc} \; & C_{mc}\left(\beta_{0},\beta_{1}\right)\\ = & \sum_{i=1}^{\infty }i\sum_{j=0}^{1}\varphi \left(i,j\right)+\sum_{i=\beta_{0}}^{\infty }\omega C_{e}\varphi \left(i,0\right)+\sum_{i=\beta_{1}}^{\infty }\omega C_{e}\varphi \left(i,1\right). \end{array}$$

Furthermore, the first term is given by

(A24) $$\begin{array}{cc} \; & \sum_{i=1}^{\infty }i\sum_{j=0}^{1}\varphi \left(i,j\right)\\ = & \sum_{i=1}^{\beta_{1}}i\varphi_{i}+\sum_{i=\beta_{1}+1}^{\beta_{0}}is_{0}^{i-\beta_{1}}+s_{0}^{\beta_{0}-\beta_{1}}\sum_{i=\beta_{0}+1}^{\infty }is_{1}^{i-\beta_{0}}\\ = & \frac{\varphi_{1}\beta_{1}\left(\beta_{1}+1\right)}{2}+\varphi_{1}A+\varphi_{1}B, \end{array}$$

where

(A25) $$A=\frac{s_{0}\left(\left(\beta_{1}+1\right)-\beta_{0}s_{0}^{\beta_{0}-\beta_{1}}\right)}{1-s_{0}}+\frac{s_{0}^{3}-s_{0}^{\beta_{0}-\beta_{1}+1}}{{\left(1-s_{0}\right)}^{2}},$$

and

(A26) $$B=\frac{\left(\beta_{0}+1\right)s_{1}}{1-s_{1}}+\frac{s_{1}^{3}}{1-s_{1}}.$$

Furthermore, the sum of last two terms is given by

(A27) $$\begin{array}{cc} \; & \sum_{i=\beta_{0}}^{\infty }\omega C_{e}\varphi \left(i,0\right)+\sum_{i=\beta_{1}}^{\infty }\omega C_{e}\varphi \left(i,1\right)\\ = & \varphi_{1}\omega C_{e}\left(s_{0}^{\beta_{0}-\beta_{1}}\sum_{i=\beta_{0}}^{\infty }s_{1}^{i-\beta_{0}}+p_{1}\sum_{i=\beta_{1}}^{\beta_{0}-1}s_{0}^{i-\beta_{1}}\right)\\ = & \varphi_{1}\omega C_{e}\left(s_{0}^{\beta_{0}-\beta_{1}}\frac{1}{1-s_{1}}+p_{1}\frac{1-s_{0}^{\beta_{0}-\beta_{1}}}{1-s_{0}}\right). \end{array}$$

This completes the proof.
